# Supplementary material for: Treatment Patterns and Outcomes by Age in Metastatic Urinary Tract Cancer: A Retrospective Tertiary Cancer Center Analysis
Source: Cancers (Basel). 2024 Jun 5;16(11):2143. doi: 10.3390/cancers16112143 (PMC11172373; doi:10.3390/cancers16112143)

Supplemental Figure S1: Kaplan-Meier analysis for PFS Treatment arm (A) comparing younger versus older patients receiving 1L chemotherapy, (B) comparing younger versus older patients receiving 1L immunotherapy. 1L= first line

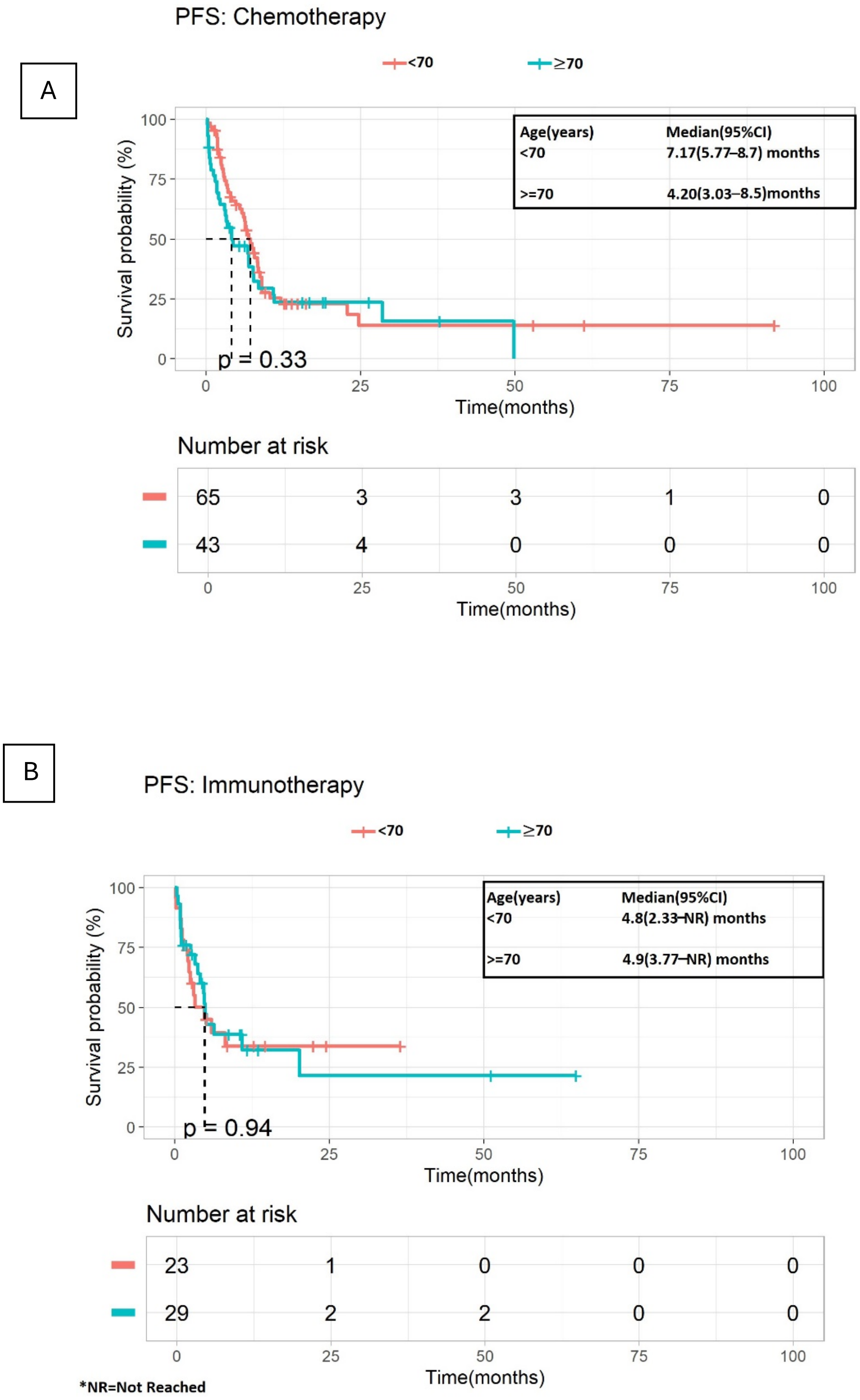

Supplement Figure S2: Kaplan-Meier analysis for (A) Overall survival by histology (B) Progression free survival by histology

A

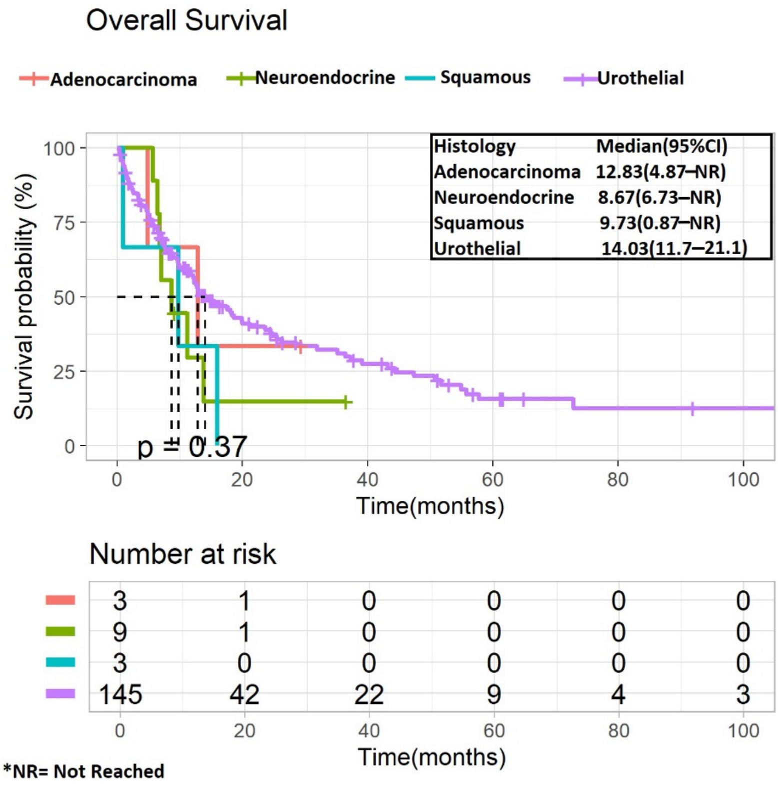

B

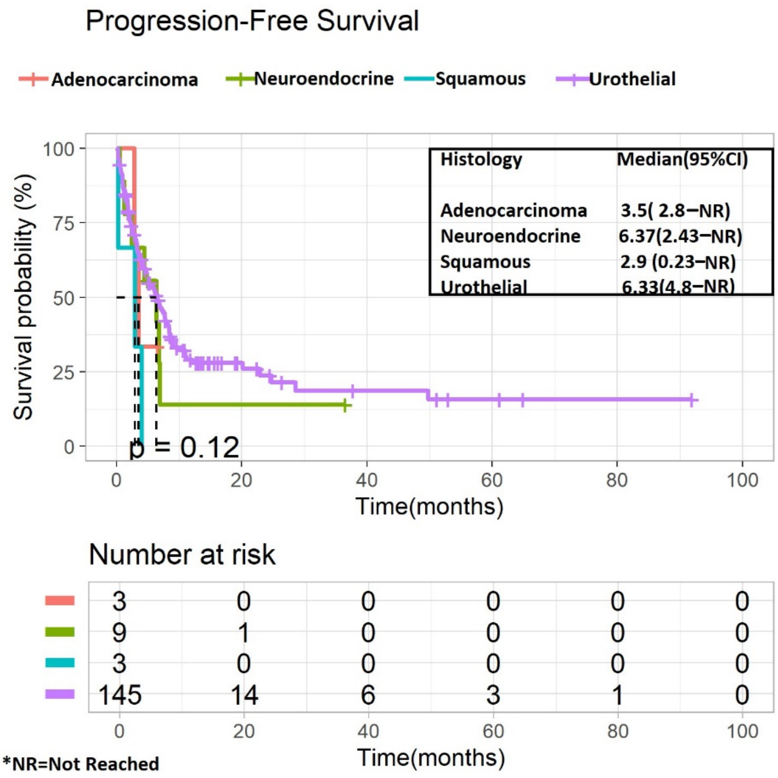

Supplement: Supplementary file 1 [file cancers-16-02143-s001.zip › cancers-2977936-supplementary.pdf]
